# Immunohistochemical Detection of *Encephalitozoon cuniculi* in Ocular Structures of Immunocompetent Rabbits

**DOI:** 10.3390/ani9110988

**Published:** 2019-11-18

**Authors:** Edita Jeklová, Lenka Levá, Vladimír Kummer, Vladimír Jekl, Martin Faldyna

**Affiliations:** 1 Veterinary Research Institute, Hudcova 296/70, 621 00 Brno, Czech Republic; jeklova@vri.cz (E.J.); leva@vri.cz (L.L.); v.kummer@seznam.cz (V.K.); 2 Jekl & Hauptman Veterinary Clinic, Mojmírovo nám. 3105/6a, 612 00 Brno, Czech Republic; vladimirjekl@gmail.com

**Keywords:** *Encephalitozoon cuniculi*, lens, eye, oral infection, immunocompetent, rabbit, encephalitozoonosis, immunohistochemistry

## Abstract

**Simple Summary:**

Encephalitozoonosis is a common infectious disease widely spread among rabbits. Its causative agent, *Encephalitozoon cuniculi*, is considered to be transmissible to humans. In rabbits, clinical signs include discoordination, head tilt, excessive water intake, excessive urination and cataracts. This study investigates, for the first time, whether the *E. cuniculi* organism can be detected in ocular structures in healthy adult rabbits after experimental oral infection using immunohistochemistry—detection of the organism in the tissue using a specific staining method. In infected animals, *E. cuniculi* spores were detected in many ophthalmic structures (periocular connective tissue, sclera, cornea, choroidea, iris, retina and lens) as early as 2 weeks after infection. There were no signs of inflammatory lesions in any of the ocular tissues examined at 2, 4, 6 and 8 weeks after infection. *E. cuniculi* was also detected in the lens of adult rabbits, which indicates that ways of lens infection other than intrauterine and haematogenic are possible. This information can help to understand *E. cuniculi* dissemination to various ocular tissues structures after oral infection.

**Abstract:**

Encephalitozoonosis is a common infectious disease widely spread among rabbits. Its causative agent, *Encephalitozoon cuniculi*, is considered as a zoonotic and emerging pathogen capable of infecting both immunocompetent and immunocompromised hosts, including humans. In rabbits, clinical signs include neurological, kidney and ocular disease. The aim of this study was to detect *E. cuniculi* in ocular structures in immunocompetent rabbits after experimental oral infection using immunohistochemistry. In infected animals, *E. cuniculi* spores were present in periocular connective tissue, sclera, cornea, choroidea, iris, retina and lens, as a round to ovoid organism reacting with a specific anti-*E. cuniculi* monoclonal antibody as early as 2 weeks after infection. There were no signs of inflammatory lesions in any of the ocular tissues examined at 2, 4, 6 and 8 weeks after infection. In the present study, *E. cuniculi* was also detected in the lenses of adult rabbits, which indicates that ways of lens infection other than intrauterine and haematogenic are possible.

## 1. Introduction

*Encephalitozoon cuniculi* is an opportunistic, obligate intracellular, single-cell, spore-forming microsporidian parasite that infects a wide range of mammalian hosts and even birds. However, the most commonly infected animals are domestic rabbits. Encephalitozoonosis was first reported in laboratory rabbits with paralysis by Wright and Craighead [1] and named by Levaditi et al. [2]. Currently, *E. cuniculi* is considered as a zoonotic and emerging pathogen capable of infecting both immunocompetent and immunocompromised hosts [3]. In humans, *E. cuniculi* has become an important opportunistic pathogen in immunosuppressed individuals, such as HIV/AIDS patients and patients receiving antitumor or immunosuppressive treatments [4,5].

Encephalitozoonosis used to be a frequent problem in laboratory rabbits, affecting the health status of the animals and interfering with experiments [6], but current research colonies are routinely tested by serological methods for specific antibodies. Nevertheless, *E. cuniculi* remains a cause of morbidity and mortality in pet and conventionally raised rabbits, with the seroprevalence of IgG antibodies in asymptomatic pet rabbits ranging between 35% and 68% [7,8,9]. 

In rabbits, horizontal transmission by ingestion or inhalation of *E. cuniculi* spores occurs most frequently [3], but intrauterine [10,11,12] and ocular infections have also been documented [13]. After ingestion, *E. cuniculi* organisms invade the intestinal epithelium and then are disseminated throughout the body via infected macrophages or by a release into the blood [14]. Organs with high blood flow such as kidneys, lungs and liver are the first target for *E. cuniculi* infection in rabbits. However, the final predilection sites are kidneys and the brain [15]. From 35 days after infection, spores are excreted intermittently in the urine [13,15]. Infected rabbits show a range of clinical signs from chronic infections, which can persist asymptomatically for years, to sudden deaths. Vestibular disease dominates among neurological signs when clinical manifestations of encephalitozoonosis occur. Kidney disease is characterised by granulomatous interstitial nephritis. 

Other predilection tissues are ocular structures. Wolfer et al. [16] suggested, that *E. cuniculi* infects the eye lens during intrauterine development, when the lens capsule is very thin or even absent and the lens has rich vascular support. Disruption of normal epithelial function could be responsible for weakness and eventual rupture of the capsule. A sudden release of lens proteins initiates the cell immune response against normal lens protein remaining in the lens, leading to phacoclastic uveitis. Besides uveitis, also cataracts of various degrees of severity can be diagnosed [17]. Nevertheless, information regarding ocular encephalitozoonosis is based mainly on the detection *E. cuniculi* in the lens of rabbits with clinically manifested phacoclastic uveitis [16,18]. 

Distribution of parasites in the lens or other ocular structures of infected immunocompetent rabbits is of interest. Therefore, the aim of this study was to detect *E. cuniculi* in ocular structures in rabbits at different time points after experimental oral infection using immunohistochemistry.

## 2. Materials and Methods

### 2.1. Preparation of E. cuniculi Spores

Spores of a rabbit strain of *E. cuniculi* (CH-K-2169; kindly provided by Prof. P. Deplazes, University of Zurich, Switzerland) were produced on the RK 13 cell line (VRI, Brno, Czech Republic) in minimal essential medium with antibiotics (10 U/mL penicillin; 0.1 mg/mL streptomycin and 0.25 μg/mL amphotericin) and 5% foetal bovine serum. The spores were harvested, resuspended in the culture medium, and stored at 4 °C. Spores were purified by density gradient centrifugation in Percoll (Sigma-Aldrich, St. Louis, MO, USA) using a standard procedure [19]. The viability of the *E. cuniculi* was verified by inoculation of an infection dose aliquot into a tissue culture.

### 2.2. Animals and Experimental Design

Outbred New Zealand White SPF European rabbits, strain Crl:KBL (free of common rabbit pathogens including *E. cuniculi*) were obtained from the Charles River Laboratories Germany. Rabbits were housed individually in wire-mesh cages in the animal care facility with controlled conditions at the Veterinary Research Institute, Brno, Czech Republic. Housing conditions were described earlier [13]. The animals were housed and handled under the agreement to protocol approved by the Branch Commission for Animal Welfare of the Ministry of Agriculture of the Czech Republic (approval project number MZe 874). 

A total of 25 four-month-old male rabbits were sedated (0.03 mg/kg medetomidine and 3 mg/kg ketamine) and then 20 rabbits were infected, using a stomach tube, with 4 × 10^7^ *E. cuniculi* spores in 1 mL of PBS and 5 control rabbits were administered only PBS. Uninfected control rabbits were housed in a separate room. After infection, rabbits were clinically monitored on a daily basis for physical activity and for any clinical signs of disease, ocular pathologies included. Based on the results of a previous experiment [13], five infected rabbits and one control rabbit were anaesthetized (0.1 mg/kg medetomidine and 15 mg/kg ketamine) and euthanised at 2, 4, 6 and 8 weeks after infection. For the purpose of this study, right eye globes of all the rabbits were collected. 

### 2.3. Immunohistochemistry

For immunohistochemistry, eye globes with periocular connective tissue were fixed in 10% neutral buffered formalin and embedded in paraffin. Serial sections (6 µm; Leica SM2000R, Leica Biosystems Nussloch GmbH, Nussloch, Germany) were mounted on silanized slides, deparaffinized in xylene (3 × 5 min), hydrated in a series of graded ethanol, and washed in Tris-HCL buffer (pH 7.6). Subsequently, heat-induced antigen retrieval was performed in a microwave on high power (750 W) for 15 min (3 × 5 min) in citrate buffer, pH 6.0. Endogenous peroxidase activity was blocked using 3% H_2_O_2_ for 15 min. The mouse anti-*E. cuniculi* monoclonal antibody (EC11C5; dilution 1:100; [20,21]) was used for the detection of *E. cuniculi*. The sections were incubated overnight at 4 °C. EnVisionTM^+^/HRP, mouse (DakoCytomation, Glostrup, Denmark) and 0.03% DAB (3,3′diaminobenzidine, Sigma-Aldrich, St. Louis, MO, USA) as a chromogen (10 min at room temperature) were used for visualization of the immunoreaction complexes. The slices were counterstained with haematoxylin. Stained sections were dehydrated and mounted under glass coverslips in Entellan (Merck, Darmstadt, Germany). Staining was performed in parallel with positive and negative controls. All the ocular structures (sclera, cornea, choroidea, iris, retina and lens) were examined in detail in multiple sections covering the entire ocular part. About 3 mm of periocular connective tissue and extraocular muscles were examined proximally to the eye globe.

## 3. Results

During the whole experimental period, orally infected rabbits did not show any signs of conjunctival, corneal or any other ocular pathologies. Overall general health status of the rabbits was also not affected at any time point after experimental infection.

**Figure 1:**
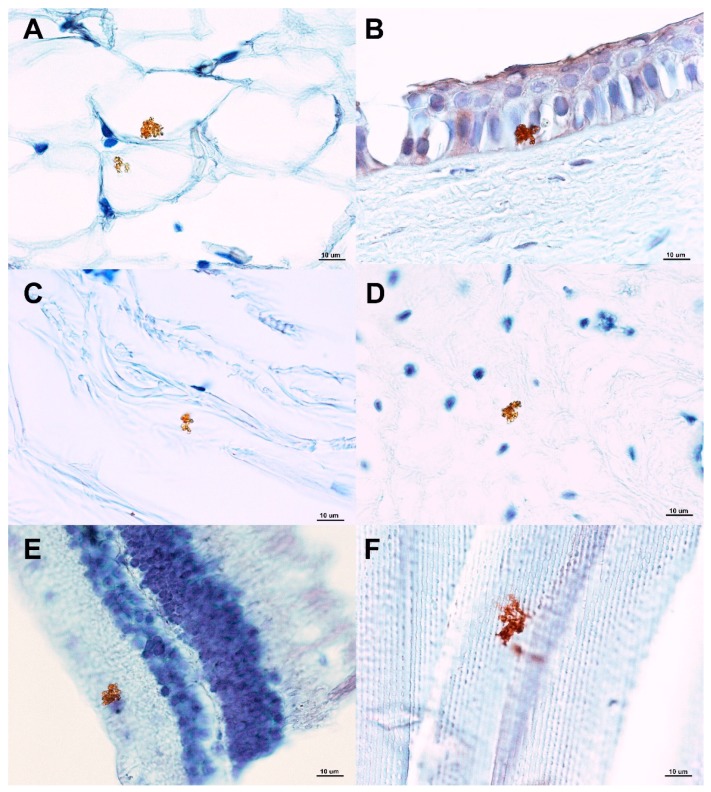
Identification of *Encephalitozoon cuniculi* using immunohistochemistry (with specific monoclonal anti-*E. cuniculi* antibody EC11C5) in various ocular structures in experimentally orally infected rabbits. Legend: (**A**) Periocular connective tissue (fat); (**B**) cornea (*E. cuniculi* detected in corneal epithelial cells); (**C**) sclera; (**D**) iris; (**E**) retina (*E. cuniculi* detected in the retinal ganglion layer); (**F**) lens (lens fibres).

Using immunohistochemistry, samples from various ocular structures of control uninfected animals did not reveal any *E. cuniculi* organism. On the contrary, in experimentally infected rabbits, *E. cuniculi* spores were present in many different ocular structures as round to ovoid organisms (2 × 2.5 µm) reacting with specific anti-*E. cuniculi* monoclonal antibody. *E. cuniculi* organisms were found to be of granular appearance and were localised intracellularly (cornea, retina) or more frequently extracellularly (Figure 1). Parasitophorous vacuoles were not observed in any of the ocular structures. No inflammatory changes of ocular tissues were detected in any of the specimens. 

Two weeks after infection, *E. cuniculi* was detected in ocular structures of 60% (3/5) of animals; 4 weeks after infection in 80% (4/5) and at 6 and 8 weeks after infection in 100% (5/5) of specimens (Table 1). 

The presence of *E. cuniculi* organisms in different ocular structures was generally low or sporadic. The most commonly affected ocular structures were sclera, cornea and periocular connective tissue. To a lesser extent, *E. cuniculi* organisms were identified in the retina, iris, lens and choroidal membrane, ciliary bodies included. The distribution of immunopositivity among particular ocular tissues is shown in Table 1.

## 4. Discussion

Encephalitozoonosis is a common infectious disease widely spread among rabbits. In immunocompetent hosts, a balanced host-parasite relationship exists. The disease is clinically silent, but parasites are present and can proliferate rapidly if immune competence is compromised. Serious lesions are usually rare or absent, except for chronic interstitial nephritis in older rabbits, which is frequently associated with subclinical, chronic, persistent infection [22]. In immunocompromised rabbits, clinical signs of neurological disorders, kidney disease or ocular lesions can be observed.

*E. cuniculi*-induced ocular lesions are described not only in rabbits but several cases of corneal microsporidiosis and microsporidial keratoconjunctivitis were also documented in immunocompetent humans [23,24,25]. Nevertheless, intraocular microsporidiosis due to *E. cuniculi* in a human patient with idiopathic CD4+ T-lymphocytopenia was also reported [26]. 

In rabbits, information related to spontaneous ocular encephalitozoonosis is based on articles describing detection of *E. cuniculi* in relation to phacoclastic uveitis [16,18,27,28]. In these cases, *E. cuniculi* was found within lens cortex, lens epithelial cells, necrotic debris, inflammatory cells and/or in lens material retrieved by phacoemulsification. No organism was detected in ocular structures other than the lens. The organism was identified based on transmission electron microscopy [16], using immunohistochemistry [27] or PCR [18,28]. Özkan and Alcigir [29] found spontaneous ocular pathology including cataract and phacoclastic uveitis in five rabbits from a total of 171 animals. However, *E. cuniculi* was detected, using Gram’s staining method, only in corneal stroma and not in the lens.

In the present study, in experimentally infected immunocompetent rabbits, *E. cuniculi* was found in many ocular structures, including periocular connective tissue, extraocular muscles, sclera, cornea, choroidea, iris, retina and lens. The microsporidia were present in all mentioned ocular structures as early as two weeks after infection, and at eight weeks after infection, *E. cuniculi* was detected in all the ocular structures of all infected rabbits.

It is still poorly understood how *E. cuniculi* gains access to the lens tissue. In adult life, the lens is an avascular segregated compartment and lens epithelial cells are completely surrounded by a thick capsule. The parasite localization within the lens thus implies a probable early infection in rabbit cubs, when the lens has a thin capsule and a rich vascular support [16,18]. During foetal development, the mammalian eye anterior chamber is partly occupied by the mesodermal tissue supported by primitive blood vessels composed of fine hyaline fibres and conjunctival cells. Hyaloid vessels and pupillary membrane (PM) form a temporary capillary network in the anterior chamber, iris diaphragm, and lens. This network nourishes the immature lens, retina, and vitreous humour during morphogenesis [30,31,32]. In the 11-day old rabbit, the PM is still functional. Functional vessels had disappeared in the centre of the pupil in the 20-day-old rabbits [32]. As *E. cuniculi* is disseminated via the blood, the lens can be directly infected by *E. cuniculi* not only in utero [11,12], but also shortly postnatally. In the present study, *E. cuniculi* was detected in the lens of adult rabbits, which indicates that other ways (apart from the haematogenic transmission) of the *E. cuniculi* lens infection may be possible. 

In previously described cases, spontaneous phacoclastic uveitis was characterized by rupture of the anterior lens capsule and the presence of neutrophils within the lens cortex [16,18]. The inflammation centered entirely around the break in the lens capsule and the anterior uveal tract was relatively unaffected. The posterior segment was also unaffected. In contrast, in the present study, inflammatory changes as well as any eye pathology were not seen within any ocular structure. These findings are in agreement with immunocompetence of experimentally infected rabbits. Moreover, in other tissues, such as brain and kidney, *E. cuniculi* spores were also frequently observed without segregating degenerative or inflammatory reaction [21,22]. 

The detection of *E. cuniculi* is usually performed by conventional histopathology, immunohistochemistry and PCR. Immunohistochemistry showed to be more sensitive than histopathological diagnosis [21]. In comparison with the PCR, immunohistochemistry allows visualisation of the exact localisation of the microorganism within the examined tissue. The specificity of the detection system was assured by using a specific mouse monoclonal antibody directed against the 45-kDa protein of *E. cuniculi*. In a previous study, this monoclonal antibody reacted with different strains of *E. cuniculi* but did not react with *E. intestinalis*, *E. hellem* and other tested bacteria or yeasts [20,21].

This original study described, for the first time, the detection of the *E. cuniculi* organism using immunohistochemistry in different ocular structures of adult immunocompetent SPF rabbits as early as two weeks after experimental oral infection. There were no signs of inflammatory lesions in any ocular tissue examined at 2, 4, 6 and 8 weeks after infection, but *E. cuniculi* was found more commonly at eight weeks after the infection. This information can help to understand *E. cuniculi* dissemination to various tissues after oral infection. In the present study, the *E. cuniculi* organism was detected in the lens of adult rabbits, which indicates that ways of lens infection other than intrauterine and haematogenic are possible. Further studies need to be performed, to confirm the pathogenesis of lens infection.

## Figures and Tables

**Table 1:** Detection rates of *E. cuniculi* in the examined ocular structures of rabbits at different time points after experimental oral infection using immunohistochemistry with specific monoclonal antibody (EC11C5).

Ocular Structure ^1^	Weeks after Infection			
	2	4	6	8
Periocular connective tissue	3	2	4	4
Extraocular muscles	2	1	2	3
Sclera	3	3	3	4
Cornea	3	3	4	4
Choroidea	2	2	3	4
Iris	1	1	2	3
Retina	1	1	3	3
Lens	2	3	3	3
No. of rabbits in which *E. cuniculi* was not detected in any ocular structure	2	1	1	0

^1^ Number of rabbits with *E. cuniculi* presence in respective ocular structure (total numbers of rabbits in respective week after infection n = 5).
